# HIV Infection Is an Independent Predictor of Mortality Among Adults with Reduced Level of Consciousness in Uganda

**DOI:** 10.4269/ajtmh.21-0813

**Published:** 2022-01-17

**Authors:** Amir A. Mbonde, Lydia Mbatidde, Bart M. Demaerschalk, Abdirahim A. Aden, Nan Zhang, Richard Butterfield, Rose Muhindo, Adrian Kayanja, Cumara B. O’Carroll

**Affiliations:** ^1^ Department of Internal Medicine, Mbarara University of Science and Technology, Uganda;; ^2^ Department of Neurology, Mayo Clinic College of Medicine and Science, Phoenix, Arizona;; ^3^ College of Science and Engineering, St. Cloud State University, New Hope, Minnesota;; ^4^ Department of Internal Medicine, Healthgate Hospital, Nairobi, Kenya;; ^5^ Department of Quantitative Health Sciences, Division of Clinical Trials and Biostatistics, Mayo Clinic, Phoenix, Arizona

## Abstract

The clinical epidemiology of adults admitted with reduced level of consciousness (LOC) in sub-Saharan Africa (SSA) and the impact of HIV infection on the risk of mortality in this population is unknown. We secondarily analyzed data from a cohort study that enrolled 359 consecutive adults with reduced LOC presenting to Mbarara Regional Hospital in Uganda with the aim of comparing the prognostic utility of the Full Outline of Unresponsiveness (FOUR) score to the Glasgow Coma Scale (GCS) Score. For this analysis, we included 336 individuals with known HIV serostatus, obtaining clinical, laboratory, and follow-up data. We recorded investigations and treatments deemed critical by clinicians for patient care but were unavailable. We computed mortality rates and used logistic regression to determine predictors of 30-day mortality. The median GCS was 10. Persons living with HIV infection (PLWH) accounted for 97 of 336 (29%) of the cohort. The 30-day mortality rate in the total cohort was 148 of 329 (45%), and this was significantly higher in PLWH (57% versus 40%, adjusted odds ratio [aOR] 2.39: 95% confidence interval [CI]: 1.31–4.35, *P *= 0.0046). Other predictors of mortality were presence of any unmet clinical need (aOR 1.72; 95% CIL 1.04–2.84, *P* = 0.0346), anemia (aOR 1.68; 95% CI: 1.01–2.81, *P* = 0.047), and admission FOUR score < 12 [aOR 4.26; 95% CI: 2.36–7.7, *P* **<** 0.0001). Presentation with reduced LOC in Uganda is associated with high mortality rates, with worse outcomes in PLWH. Improvement of existing acute care services is likely to improve outcomes.

## INTRODUCTION

The spectrum of underlying diseases associated with reduced LOC in Uganda and sub-Saharan Africa (SSA) is broad and distinctly different when compared with that of patients seen in other parts of the world. Central nervous system (CNS) infections, severe sepsis from a non-CNS infection, and metabolic encephalopathy are the leading causes of reduced LOC in SSA.^1^^–^^4^ Outcomes in this population are generally poor, with estimated mortality rates of approximately 40% to 60%.^1^^,^^2^

HIV infection, which is highly prevalent in the region, appears to be a common underlying contributing factor for most patients with reduced LOC. A retrospective review of the causes of admission and death among all adult medical admissions in Uganda showed that PLWH accounted for 30% and 25% of all in-patient admissions and fatalities, respectively.^5^ PLWH in SSA account for 10% to 30% of all admitted adult individuals with reduced LOC.^4^^,^^6^ HIV infection leads to reduced LOC either through the direct effects of the virus on the CNS or, perhaps most commonly, through immunosuppression, thus increasing one’s vulnerability to opportunistic CNS infections.^7^^–^^10^ In addition, impaired immune defense mechanisms secondary to HIV infection predisposes patients to worse forms of any systemic disease, eventually leading to reduced LOC.^11^ Despite the strong association between HIV infection and reduced LOC, its impact on mortality among adult individuals with reduced LOC in SSA is unclear. Furthermore, most hospitals in SSA are faced with the challenge of a dire lack of adequate human and material resources,^12^ which might also lead to poor outcomes.

In this study, we aimed to describe the clinical epidemiology of adults presenting with reduced LOC to a tertiary referral hospital in a high HIV endemic setting in southwestern Uganda. We assessed for predictors of mortality while evaluating the impact of HIV infection and inadequate resource availability (unmet clinical needs) on mortality. We aimed to provide highly impactful clinical information that can be used by clinicians taking care of patients in SSA to directly improve patient outcomes. Additionally, establishing a direct association between unmet clinical needs and poor outcomes could potentially stimulate discussions among policy makers and providers, geared toward the derivation of innovative, low-cost interventions that could result in improved patient outcomes.

## MATERIALS AND METHODS

### Study design and study setting.

This was a secondary analysis of data derived from a cohort study that enrolled 359 consecutive adults with reduced LOC of any cause, admitted to the medical ward of Mbarara Regional Referral Hospital (MRRH) in southwestern Uganda between April 2017 and April 2018.^13^ The main aim of the primary study was to compare the prognostic utility of the Full Outline of Unresponsiveness (FOUR) score to the Glasgow Coma Scale (GCS) score.^13^

MRRH is a tertiary public institution that serves as the teaching hospital for Mbarara University of Science and Technology and has a catchment population of more than 2 million. Initial assessment and treatment of all critically ill adult patients are conducted at the medical emergency department. Most patients who stabilize after initial treatment are admitted to the general medical ward. Those who remain unstable or critically ill are admitted to the high monitoring unit, which acts as a step-down unit, or an adjacent 10-bed capacity intensive care unit (ICU). Only a small proportion of critically ill individuals are admitted to the ICU due to its limited bed capacity.

MRRH offers free basic investigations such as complete blood count, basic chemistry, random blood sugar, HIV test, tuberculosis tests (Ziehl Nielson and GeneXpert studies), chest X-ray, electrocardiography, malaria testing, and urinalysis. All other additional investigations, such as more extensive blood tests, computed tomography (CT) scan, echocardiography, and ultrasound scanning, are only available to those who can afford to pay for the service. With regard to treatment, critically ill individuals admitted to MRRH can access basic resuscitative treatments such as intravenous fluids, steroids, insulin, antimalarial medications, oxygen therapy, and a limited range of antibiotics such as ceftriaxone, gentamicin, and benzylpenicillin. All other prescribed medications beyond what is available on the hospital formulary are only administered if the patient can purchase them from a private pharmacy.

### Inclusion and exclusion criteria.

For this secondary analysis, we included all patients previously enrolled in our prior cohort of individuals with reduced LOC regardless of the underlying etiology.^13^ The primary study excluded all patients whose primary cause of reduced LOC was determined to be surgical. We excluded patients with unknown HIV status.

### Study procedures and patient follow-up.

A detailed description of the study procedures is available in our previous publication.^13^ All adult individuals with reduced LOC were initially identified and screened by our study team, and informed consent was obtained if the patient met inclusion criteria. Reduced level of consciousness was defined as either being alert but not fully oriented or not being alert. All participants were evaluated by one of our study team physicians, which consisted of a hospitalist and two senior internal medicine residents. The study team assessments were only conducted after the hospital physicians had completed their assessment and initial treatment had been administered. One of the study physicians subsequently conducted a detailed clinical exam and completed a study questionnaire. All participants were followed throughout their hospitalization. Treatments prescribed and administered by the hospital providers were recorded without any direct intervention from our study team. We recorded any unmet clinical need encountered. We defined an unmet clinical need as any treatment or medication that the hospital providers deemed to be necessary for a particular patient’s care but the treatment or test was either unavailable at the public hospital or could not be privately procured (afforded) by the patient. To obtain information on the unmet clinical need, we reviewed patient charts for tests or medications that had been prescribed/ordered but not administered due to lack of availability. This was done at discharge or death. The hospital providers had to have documented the test or medication in the plan of care in anticipation that the test or medication was either available or that the patient could afford to buy it if unavailable. We also took note of situations in which providers documented in the assessment and plan section that a particular test or medication would have been ideal but was unavailable.

The study did not perform head CT scans for enrolled participants. Head CT scans are typically paid for by patients, their family members, or well-wishers. If this test was ordered in the chart but not completed (or if written in the assessment and plan as an ideal test that was not available), then this was recorded as an unmet clinical need.

All participants were followed for the entire length of their hospitalization. Any in-hospital complications that arose during the hospitalization, defined as the occurrence of an adverse event such as status epilepticus, acute hypoxic respiratory failure, severe hypotension, need for mechanical ventilation, worsening coma, for example, were noted. Those discharged alive from the hospital were followed up via a phone call to determine their survival status at 30 days.

### Final diagnosis.

We recorded the final diagnoses directly from the patient’s chart as noted by the hospital physicians. We classified the final diagnoses into metabolic encephalopathy, CNS infection, severe sepsis from non-CNS infections, seizures, stroke, and other diagnosis (Table 1).^13^ Metabolic encephalopathy was defined as reduced LOC primarily resulting from metabolic abnormalities such electrolyte derangements, glucose derangements, liver failure, renal failure, or hypoxia. Examples of CNS infection pathologies included tuberculous meningoencephalitis, toxoplasmosis, fungal meningoencephalitis, cerebral malaria, acute viral meningitis, and acute bacterial meningitis. Examples of non-CNS infections were pneumonia, disseminated tuberculosis, and urinary tract infections. All participants with stroke had a CT head scan before inclusion. Diagnoses considered under “other” included brain metastases, brain tumor, suspected hypoxic ischemic encephalopathy, and unknown.

**Table 1:** Baseline characteristics of all participants, stratified by HIV status

Variable	HIV infected (*n* = 97)	HIV uninfected (*n* = 239)	Total cohort (*n* = 336)	*P* value
Age, mean (SD)	39 (13)	56 (23)	51 (22)	< 0.0001*
Male, *n* (%)	51 (53)	144 (60)	195 (58)	0.1965†
Presenting symptoms, *n* (%)				
Headache	34 (35)	56 (23)	90 (27)	0.0293†
Vomiting	28 (29)	37(16)	65 (19)	0.0049†
Diarrhea	19 (20)	8 (3)	27 (8)	< 0.0001†
Fever	20 (21)	37 (16)	57 (17)	0.2555†
Cough	28 (29)	25 (11)	53 (16)	< 0.0001†
Poisoning	3 (3)	15 (6)	18 (5)	0.2954‡
Physical signs				
Abnormal chest auscultation findings, *n* (%)	33/95 (35)	98/237 (41)	131 (40)	0.2652†
Respiratory rate, breath/min, median IQR	22 (20, 30)	22 (19, 28)	22 (20, 28)	0.2101^1^
Admission FOUR Score, median (IQR)	14 (12, 15)	13 (11, 15)	14 (11, 15)	0.5339*
Glasgow Coma Scale, median (IQR)	11 (8, 13)	10 (8, 13)	10 (8, 13)	0.4263*
Laboratory findings				
Leucopenia (WBC < 11 cells/mm^3^)	74 (76)	136 (57)	210 (63)	0.0009†
Anemia (Hb < 12 g/dL)	56 (58)	77 (32)	133 (40)	< 0.0001*
Serum sodium < 135 mmol/L	45 (46)	96 (40)	141 (42)	0.2948†
Serum potassium < 3.5 mEq/L	70 (72)	160 (67)	230 (69)	0.3509†
Serum creatinine ≥ 1, mg/dL, *n* (%)	47 (49)	127 (53)	162 (48)	0.4361†
Lumbar puncture performed, *n* (%)	34 (35)	24/238 (10)	58/335 (17)	< 0.0001†
Final diagnosis *n* (%)				< 0.0001‡
Metabolic encephalopathy	27 (28)	79 (33)	106 (32)	
CNS infection	44 (45)	49 (20)	93 (28)	
Stroke	5 (5)	68 (28)	73 (22)	
Severe sepsis from a non-CNS infection	17 (18)	19 (8)	36 (11)	
Seizures	2 (2)	10 (4)	12 (4)	
Other diagnosis	2 (2)	14 (6)	16 (5)	

CNS = central nervous system; FOUR = Full Outline of Unresponsiveness subscale; Hb = hemoglobin; IQR = interquartile range; SD = standard deviation; WBC = white blood cell count.

* Kruskal-Wallis *P* value.

† Chi-square *P* value.

‡ Fisher exact *P* value.

### Study outcome.

The primary outcome for this study was mortality rate at 30 days. Other outcomes evaluated included in-hospital mortality and the occurrence of any in-hospital complications, such as respiratory failure, status epilepticus, or worsening LOC.

### Data collection and statistical analysis.

Data was collected using a pretested questionnaire and later entered into the REDCap database hosted on the Mayo Clinic server. We summarized the baseline characteristics of all participants such as sociodemographics, and other clinical parameters into either proportion, median (interquartile range [IQR]), or mean (standard deviation [SD]). Demographics, baseline clinical characteristics, and unmet clinical needs were compared between HIV-infected patients and HIV-uninfected patients using Kruskal-Wallis, chi-square, or Fisher’s exact test where applicable. We computed mortality rates and used logistic regression to determine univariate predictors of 30-day mortality. On the basis of univariate significance level cutoff of *P* ≤ 0.1, a multivariable logistic model was built to determine predictors of 30-day mortality. We did not include in the multivariate model any two variables that were correlated. For example, lack of cardiorespiratory support and lack of medication as unmet clinical needs were not entered in the final model because they were highly correlated with presence of any unmet clinical need. The GCS score was also highly correlated with the FOUR score (Spearman correlation coefficient 0.877, *P* < 0.0001) and hence only FOUR score was included into the multivariate model. Kaplan-Meir method and log-rank test were used to estimate and compare 30-day survival between HIV uninfected and PLHIV univariately.

### Ethical considerations.

All study participants or their caregivers provided informed consent before enrollment. The research ethics committees of Mbarara University of Science and Technology and the Mayo Clinic in Arizona approved the study.

## RESULTS

### Baseline characteristics in the total cohort.

The baseline sociodemographic, clinical, and laboratory characteristics of all participants are summarized in Table 1. We analyzed data from all 336 of 359 patients enrolled in the original study after excluding 23 of 359 with unknown HIV serostatus.^13^ Excluded individuals (*n* = 23) had baseline characteristics that were comparable to those of included individuals (*n* = 336) (Supplemental Table 1).

Of those analyzed, 29% (97 of 336) had HIV infection, and more than half were men, 58% (195 of 336). The median (IQR) admission FOUR score and GCS score were 14 (11–15) and 10 (8–13), respectively, in the total cohort. Metabolic encephalopathy was the most common final hospital diagnosis, accounting for 32% (106 of 336) of all patients, followed by CNS infections (28%, 93 of 336), stroke (22%, 73 of 336), and severe sepsis from non-CNS infections (11%, 36 of 336) (Table 1).

Hyponatremia (serum sodium < 135 mmol/L), hypokalemia (serum potassium < 3.5 mmol/L) and elevated creatinine (serum creatinine > 1 mmol/L) were seen in 42% (141 of 336), 69% (230/336) and 48% (162/336) of participants, respectively.

### Baseline characteristics stratified by HIV status.

PLWH subjects were younger than their HIV-uninfected counterparts with a mean (SD) age of 39 years (13) versus 56 years (23) (*P* < 0.0001). PLWH compared with HIV-uninfected subjects were more likely to present with diarrhea (20%, 19 of 97 versus 3%, 8 of 239, *P < *0.0001), vomiting (29%, 28 of 97 versus 16%, 37 of 239, *P *= 0.0049), headache (35%, 34 of 97 versus 23%, 56 of 239, *P = *0.0293), and cough (29%, 28 of 97 versus 11%, 25 of 239 *P* ≤ 0.0001). Physical signs such as median FOUR Score, coma (GCS < 9), any abnormal chest auscultatory signs (crepitations, consolidations, effusions), and respiratory rate were similar between the two study groups (Table 1). PLWH compared with their HIV-uninfected counterparts were more likely to present with anemia (58%, 56 of 97 versus 32%, 77 of 239, *P *< .0001) and leukopenia (76%, 74/97 versus 57%, 136/239, *P *= 0.0009), although other laboratory tests such as serum potassium, serum sodium, and serum creatinine were comparable (Table 1). Lumbar punctures were more frequently performed in PLWH compared with HIV-uninfected subjects (35%, 34 of 97 versus 10%, 24 of 238, *P *< 0.0001). Regarding the final diagnosis, PLWH commonly presented with CNS infections (45%, 44 of 97 versus 20%, 49 of 239) and other non-CNS infections (18%, 17 of 97 versus 8%,19 of 239). In contrast, HIV-uninfected subjects were more likely to present with metabolic encephalopathy (33%, 79 of 239 versus 28%, 27 of 97) and stroke (28%, 68 of 239 versus 5%, 5 of 97, *P *< 0.0001).

### Unmet clinical need.

An unmet clinical need was encountered in 45% (150/336) of the total cohort (Table 2). However, this finding was disproportionately more common in PLWH compared with HIV-negative individuals (55%, 53 of 97 versus 41%, 97 of 239, *P *= 0.0189). In terms of specific unmet clinical needs, PLWH were comparatively more likely to encounter an unmet clinical need for additional laboratory testing (26%, 25 of 97 versus 9%, 22 of 239, *P *< 0.0001). The rates of other specific unmet clinical needs such as lack of medication, lack of ICU bed availability, and lack of head imaging were similar between the two groups.

**Table 2:** Unmet clinical need and outcomes, stratified by HIV status

	HIV infected (*n* = 97)	HIV uninfected (*n* = 239)	Total cohort (known HIV) *n* = 336	*P* value
Unmet clinical needs, *n* (%)				
None	44 (45)	142 (59)	186 (55)	0.0189†
Any unmet clinical need	53 (55)	97 (41)	150 (45)	
Laboratory testing	25 (26)	22 (9)	47 (14)	< 0.0001†
Medication	10 (10)	14 (6)	24 (7)	0.1511†
Cardiorespiratory support/ICU	15 (16)	50 (21)	65 (19)	0.2512†
Head Imaging or EEG	4 (4)	20 (8)	24 (7)	0.2423‡
Outcomes				
Occurrence of any in-hospital complication, *n* (%)	54/83 (65)	93/206 (45)	147/289 (51)	0.0022†
30-day mortality, *n* (%)	55/96 (57)	93/233 (40)	148/329 (45)	0.0040†

EEG = Electroencephalogram; ICU = intensive care unit.

* Kruskal-Wallis *P* value.

† Chi-square *P* value.

‡ Fisher exact *P* value.

### Outcomes.

**Figure 1.**
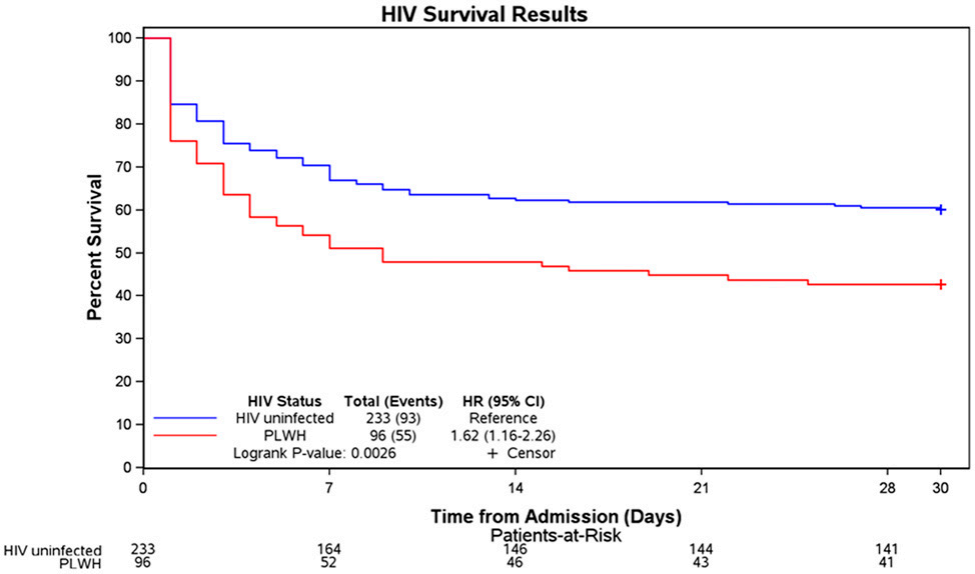
Kaplan-Meier curves showing survival according to HIV serostatus. HR = hazard ratio; PLWH = people living with HIV. PLWH had a significantly lower 30-day mortality rate compared with HIV-uninfected individuals (log-rank value = 0.026). This figure appears in color at www.ajtmh.org.

With regard to outcomes (Table 2), seven individuals were lost to follow-up. The 30-day mortality rate in the total cohort was 45% (148 of 329) and this was significantly higher in PLWH, 57% (55 of 96) compared with HIV-uninfected individuals, 40% (93 of 233, *P = *0.0040). This finding on the 30 day mortality rate being higher in PLWH is further demonstrated on the Kaplan-Meier survival curves, log-rank P value of 0.026 (Figure 1). In-hospital complications were seen in 51% (147 of 289) of all those in whom this variable was available. PLWH more commonly encountered an in-hospital complication compared with their HIV-uninfected counterparts (65%, 54 of 83 versus 45%, 93 of 206, *P = *0.0022).

### Predictors of 30-day mortality.

Significant predictors of 30-day mortality (Table 3) on multivariate analysis were as follows: presence of any unmet clinical need (adjusted odds ratio [aOR] 1.72; 95% confidence interval [CI]: 1.04–2.84, *P *= 0.0346), presence of HIV infection (aOR 2.39; 95% CI: 1.31–4.35, *P = *0.0046), presence of anemia (aOR 1.68; 95% CI: 1.01–2.81, *P *= 0.047), and admission FOUR Score < 12 (aOR 4.26; 95% CI: 2.36–7.7, *P *< .0001). Individuals with pesticide poisoning were unlikely to die (aOR 0.24; 95% CI: 0.06–0.94, *P *= 0.0410).

**Table 3:** Univariate and multivariable logistic results for 30-day mortality

Variable		Univariate		Multivariable	
	Reference	OR (95% CI)	*P* value*	OR (95% CI)	*P* value*
Age	1-unit increase	1.003 (0.993–1.013)	0.5387	1.01 (1–1.03)	0.0607
Abnormal chest findings on auscultation	Yes vs. no	2.18 (1.39–3.42)	0.0007	1.58 (0.94–2.65)	0.0864
Dyspnea	Yes vs. no	1.93 (0.96–3.88)	0.0636	1.26 (0.57–2.78)	0.5657
Fever	Yes vs. no	0.98 (0.55–1.75)	0.9550		
Headache	Yes vs. no	1.16 (0.71–1.90)	0.5598		
Vomiting	Yes vs. no	1.99 (1.14–3.47)	0.0158	1.69 (0.9–3.18)	0.1035
Diarrhea	Yes vs. no	1.35 (0.61–2.97)	0.4554		
Ingestion of a poisonous pesticide	Yes vs. no	0.23 (0.06–0.81)	0.0218	0.24 (0.06–0.94)	0.0410
Presence of any unmet clinical need	Yes vs. no	2.00 (1.29–3.11)	0.0021	1.72 (1.04–2.84)	0.0346
Unmet clinical need					
Additional laboratory testing	Yes vs. no	0.79 (0.42–1.50)	0.4701		
Medications/treatment	Yes vs. no	3.78 (1.45–9.86)	0.0065		
Cardiorespiratory support	Yes vs. no	3.52 (1.97–6.29)	< 0.0001		
Brain imaging or EEG	Yes vs. no	0.51 (0.20–1.28)	0.1522		
Presence of anemia	< 12 vs. ≥ 12	1.91 (1.22–2.99)	0.0045	1.68 (1.01–2.81)	0.0471
HIV infection status	Positive vs. negative	2.02 (1.25–3.27)	0.0043	2.39 (1.31–4.35)	0.0046
Serum potassium	≥ 3.5 vs. < 3.5	1.24 (0.77–2.00)	0.3773		
Respiratory rate	≥ 22 vs. < 22	1.37 (0.88–2.12)	0.1649		
Admission FOUR score	< 12 vs. ≥ 12	3.49 (2.10–5.79)	< 0.0001	4.26 (2.36–7.7)	< 0.0001
Admission GCS score	< 9 vs. ≥ 9	3.83 (2.30–6.38)	< 0.0001		

CI = confidence interval; EEG = electroencephalogram; FOUR = Full Outline of Unresponsiveness subscale; GCS = Glasgow Coma Scale; OR = odds ratio.

## DISCUSSION

To our knowledge, our study offers the first comprehensive real-world description of the clinical epidemiology, treatment challenges, and outcomes of individuals with reduced LOC admitted to a public academic hospital in a high HIV endemic setting in SSA. The mortality rate was high in our cohort, consistent with findings from other prior studies within the region,^1^ but significantly lower than findings from high-income countries.^14^

Nearly one in every three individuals in our cohort had HIV infection. PLWH with reduced LOC were younger than their HIV-uninfected counterparts and more frequently presented with gastrointestinal (GI) symptoms, headache, leukopenia, and anemia suggesting the presence of immunosuppression, opportunistic infections, and AIDS. Not surprisingly, PLWH were commonly diagnosed with either a CNS or non-CNS infection. Our findings demonstrate that despite the widespread rollout of highly active antiretroviral therapy in SSA, PLWH continue to present to hospitals with complications attributable to immunosuppression/AIDS. Diarrheal illnesses such as viral gastroenteritis, giardiasis, and tuberculosis are prevalent in PLWH with AIDS.^15^ It will therefore be interesting to study how the epidemiology of adult individuals with reduced LOC in Uganda evolves with the success of the ongoing “HIV test and treat campaign” in SSA.^16^

HIV infection was a strong risk factor for mortality in our study and, not surprisingly, can be directly linked to the disproportionately increased number of infections and severe sepsis in PLWH in the cohort. CNS and systemic infections including tuberculosis meningitis, bacterial meningitis, toxoplasmosis, and cryptococcal meningitis are traditionally associated with high mortality rates among PLWH in SSA.^17^^,^^18^ Another explanation for the higher rates of death in PLWH in our study is the disproportionately high rates of having an unmet clinical need. PLWH were significantly more likely to have needed an additional critical laboratory test that was not available. These unavailable studies might have included expanded microbiological studies beyond what was available, histopathological studies, and abdominal-pelvic-chest imaging aimed at evaluating for a targetable and potentially treatable source of infection. HIV is a multisystemic disease that affects many organs with patients potentially presenting with various complications that may require a more comprehensive workup.^19^ Furthermore, PLWH are likely to be unemployed^20^ or of a lower socioeconomic status and thus unable to afford required medications or investigations that are not freely available at the public hospital. PLWH also tend to have higher readmission rates^21^ and therefore likely to be in and out of hospitals, which not only affects their ability to earn a living and cover hospital costs, but also puts them at a heightened risk of adverse effects related to multiple hospitalizations.

Overall, participants in our study who had any kind of unmet clinical need such as lack of a prescribed medication, need for intensive care, need for diagnostic head imaging, or additional laboratory testing were at higher odds of death. We have thus demonstrated an association or, more likely, a direct causal relationship between inadequate hospital resources and poor outcomes in Uganda. An unmet clinical need adversely affects patient outcomes in a multitude of ways. Individuals with systemic infection and sepsis who do not receive adequate and timely administration of appropriate antibiotics and fluids have been shown to have poor outcomes in Uganda.^22^ Therefore, recent efforts to prioritize and increase resource allocation to hospitals in austere settings such as ours will directly lead to improved outcomes in adult patients with reduced LOC.^23^^,^^24^ Individuals with respiratory distress may require a complete respiratory work-up and close monitoring in the ICU, but this may not always be available. The lack of adequate intensive care facilities to offer cardiorespiratory support to those with respiratory distress and respiratory failure in Uganda is undoubtedly associated with mortality. Approximately 18% of individuals in our cohort did not have access to the ICU when they required it. A recent survey of ICU capacity in Uganda found that there were fewer than 100 functional ICU beds in the entire country, giving an estimated ratio of 1.3 ICU beds per million populations,^25^ and thus not all those who require these critical care services have access, which most certainly contributes to poor outcomes. Uganda is currently in the midst of the COVID-19 pandemic that has placed a significant strain on the already meager ICU resources available.

Interestingly, we found that patients presenting with reduced LOC secondary to the ingestion of pesticides were unlikely to die. Further research aimed at understanding this intriguing finding is warranted. Anecdotally, we have noted that individuals who ingest poisons are more likely to be brought to the hospital sooner. They are also most likely to receive prehospital first aid treatment, which might include the induction of emesis to promote excretion of the ingestant. Treatment interventions offered at the hospital such as activated charcoal, atropine, and nasogastric tube lavage are often available and effective.

This was a secondary analysis of previously collected data, and therefore some key explorative variables needed for this analysis were not available. For example, information on HIV-specific variables such as antiretroviral status, CD4 status, viral load, and duration of HIV infection were not available to allow for subgroup analyses and further exploration of the poor outcomes seen in this group. Nevertheless, the study provides preliminary data on which future studies can be designed to better understand the factors associated with mortality in these patients. Lack of adequate testing meant that some of the patient diagnoses were not confirmed or potentially miscategorized. However, we recorded the actual diagnosis that the patients were being treated for by the hospitalists, thus providing a real-world, observational assessment of individuals with reduced LOC in Uganda

HIV infection is common among adults hospitalized with reduced LOC in Uganda and is associated with an elevated risk of mortality. PLWH in our cohort were younger than their HIV-uninfected counterparts and more frequently presented with symptoms and signs suggestive of severe immunosuppression and AIDS. Other predictors of mortality included a low FOUR score, presence of any unmet clinical need, and anemia. Targeted management geared toward evaluating and treating some of these complications and increased healthcare resource allocation may lead to better outcomes.

## Supplemental Material


Supplemental materials
