# Supplementary material for: HIV Infection Is an Independent Predictor of Mortality Among Adults with Reduced Level of Consciousness in Uganda
Source: Am J Trop Med Hyg. 2022 Jan 17;106(3):909–14. doi: 10.4269/ajtmh.21-0813 (PMC8922480; doi:10.4269/ajtmh.21-0813)
Supplement: Supplementary file 1 [file tpmd210813.SD1.pdf]

SUPPLEMENTAL TABLE 1

Comparison between individuals included and excluded from the current analysis.

Variable	Included (*N* = 336)	Excluded (*N* = 23)	*P* value
Age, mean (SD)	50.8 (21.7)	57.8 (26.2)	0.2014*
Male, *n* (%)	195 (58.0%)	15 (65.2%)	0.4989†
Presenting symptoms, *n* (%)			
Headache	90 (26.8%)	5 (21.7%)	0.5956†
Vomiting	65 (19.3%)	6 (26.1%)	0.4323†
Diarrhea	27 (8.0%)	2 (8.7%)	0.9105†
Fever	57 (17.0%)	6 (26.1%)	0.2658†
Cough	53 (15.8%)	1 (4.3%)	0.1381†
Poisoning	18 (5.4%)	4 (17.4%)	0.0199†
Physical signs			
Abnormal chest auscultation findings, *n* (%)	131 (39.5%)	7 (30.4%)	0.3906†
Respiratory rate, breaths/min, median IQR	22 (20–28)	21 (20–32)	0.8831*
Admission FOUR score, median (IQR)	14 (11–15)	12 (10–15)	0.4009*
Glasgow Coma Scale, median (IQR)	10 (8–13)	10 (7–11)	0.3450*
Laboratory Findings			
Leucopenia (WBC < 11 cells/mm^3)	210 (62.5%)	13 (56.5%)	0.5675†
Anemia (Hb < 12 g/dL)	133 (39.6%)	7 (30.4%)	0.3842†
Serum sodium < 135 mmol/l	141 (42.0%)	13 (56.5%)	0.1724†
Serum potassium < 4.5 mEq/L	100 (29.8%)	7 (30.4%)	0.9456†
Serum creatinine ≥ 1, mg/dL, *n* (%)	174 (51.8%)	12 (52.2%)	0.9712†
Lumbar puncture performed, *n* (%)	58 (17.3%)	2 (8.7%)	0.2844†
Final diagnosis *n* (%)			0.8500†
Metabolic encephalopathy	106 (31.5%)	8 (34.8%)	
Central nervous system infections	93 (27.7%)	5 (21.7%)	
Stroke	73 (21.7%)	7 (30.4%)	
Severe sepsis from a non-CNS infection	36 (10.7%)	2 (8.7%)	
Seizures	12 (3.6%)	0 (0.0%)	
Other diagnosis	16 (4.8%)	1 (4.3%)	

CNS = central nervous system; FOUR = Full Outline of Unresponsiveness subscale; Hb = hemoglobin; IQR = interquartile range; SD = standard deviation; WBC = white blood cell count.

* Kruskal-Wallis *P* value.

† Chi-square *P* value.

‡ Fisher exact *P* value.
